# Supplementary material for: Federated unsupervised random forest for privacy-preserving patient stratification
Source: Bioinformatics. 2024 Sep 4;40(Suppl 2):ii198–207. doi: 10.1093/bioinformatics/btae382 (PMC11373406; doi:10.1093/bioinformatics/btae382)
Supplement: btae382_Supplementary_Data [file btae382_supplementary_data.zip › uRF_Supplement.pdf]

Supplementary Material
Federated unsupervised random forest for privacy-preserving patient stratification

March 11, 2024

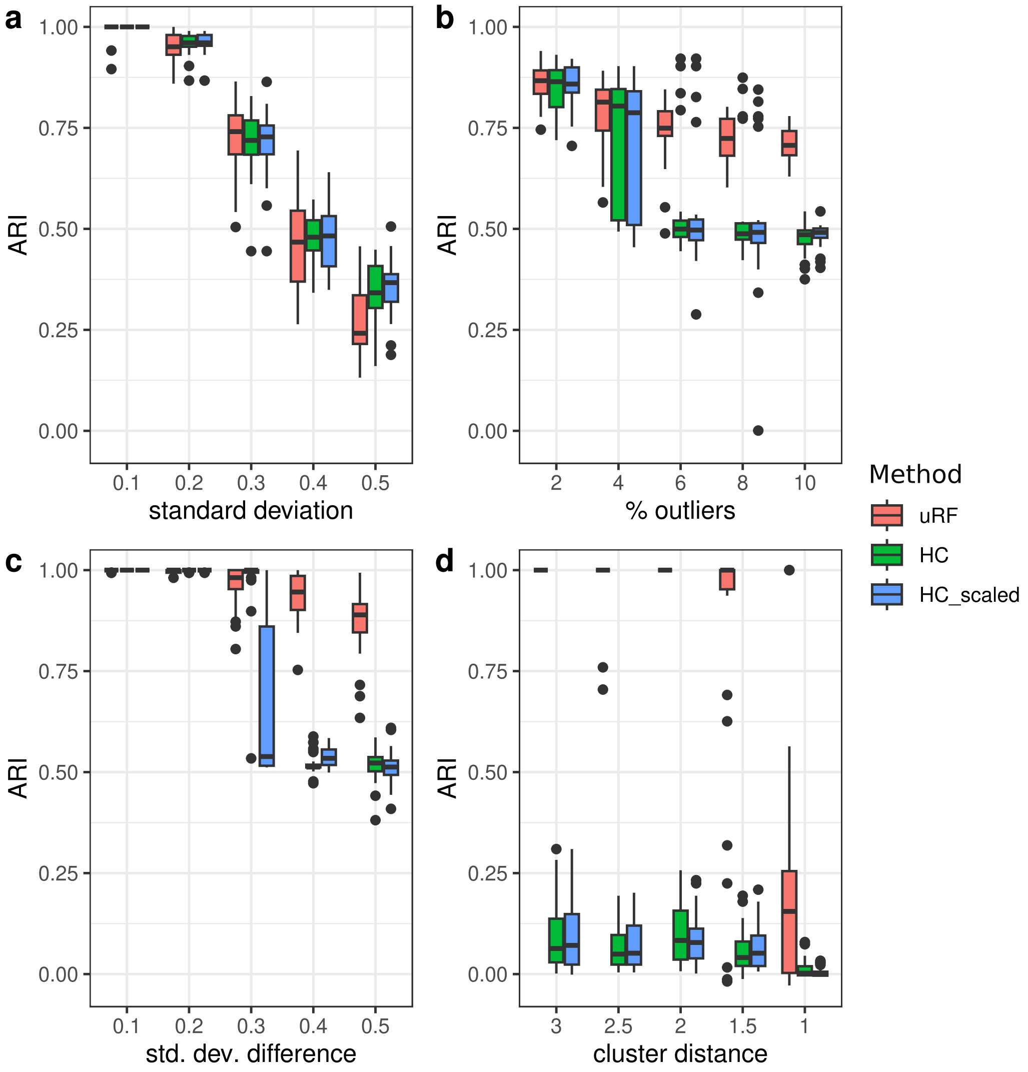

Figure 1: Clustering performance in terms of Adjusted Rand Index (ARI) of the Ward clustering algorithm on affinity matrices derived from the proposed unsupervised Random Forest with novel split rule *uRF*, Euclidean distance (HC) and Euclidean distance on standardised data (HCscaled), evaluated in four scenarios: (a) globular clusters of equal size, (b) globular clusters with outliers, (c) globular clusters of varying sizes, and (d) non-globular clusters shaped as concentric circles.

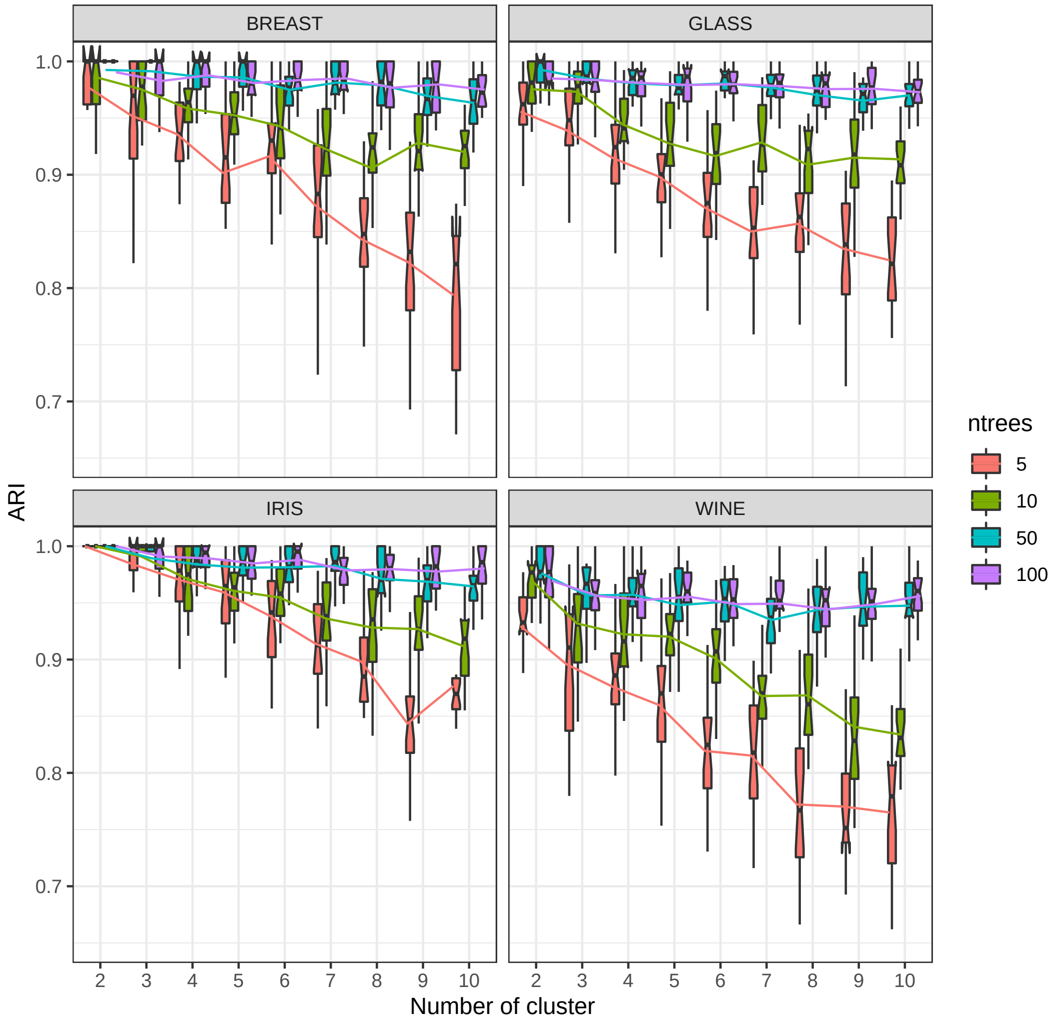

Figure 2: Verifying the optimal number of clusters using the proposed unsupervised random forest by subsequently reducing the number of trees. The clustering solutions at varies levels of k were created using an unsupervised random forest comprising 500 trees. The derived affinity matrix served as an input for hierarchical clustering. The dendrogram was cut at different k levels and the resulting multi-class labels were passed back to the unsupervised random forest as a response vector. In this way we label the samples within the leaf nodes of the unsupervised random forest to allow for predictions.
